# Efficacy and Safety of Psychedelics in Mental Disorder Cases: An Umbrella Review of Meta-Analyses of Randomized Controlled Trials

**DOI:** 10.3390/jcm15010253

**Published:** 2025-12-29

**Authors:** Monika Dominiak, Adam Gędek, Szymon Modrzejewski, Agnieszka Permoda-Pachuta, Anna Zofia Antosik

**Affiliations:** 1 Institute of Psychiatry and Neurology, 02-957 Warsaw, Poland; 2 Third Department of Psychiatry, Institute of Psychiatry and Neurology, 02-957 Warsaw, Poland; 3 Faculty of Medicine, Medical University of Lublin, 20-059 Lublin, Poland; 4 Department of Neuroses, Personality Disorders and Eating Disorders, Institute of Psychiatry and Neurology, 02-957 Warszawa, Poland; 5 Department of Psychiatry, Faculty of Medicine, Collegium Medicum, Cardinal Wyszynski University in Warsaw, 01-938 Warszawa, Poland

**Keywords:** psychedelics, psilocybin, depression, MDMA, PTSD, post-traumatic stress disorder, alcohol use disorder

## Abstract

**Background**: Psychedelic-assisted therapy is gaining renewed attention as a potential treatment for various mental disorders. Despite increasing numbers of randomized controlled trials (RCTs) and meta-analyses, a comprehensive synthesis of the evidence across different substances and indications is lacking. This umbrella review aims to evaluate the effectiveness and safety of psychedelic-assisted therapy—primarily psilocybin, MDMA, and LSD—across major psychiatric disorders, including depression, post-traumatic stress disorder (PTSD), and substance use disorders. **Methods**: We systematically identified and synthesized data from 23 meta-analyses encompassing over 100 primary studies. Outcomes were standardized and re-expressed as Hedges’ g to enable cross-study comparisons. Study quality was assessed using AMSTAR2, and certainty of evidence was evaluated via the GRADE framework. **Results**: The number of identified meta-analyses differed markedly depending on the substance and clinical indication: psilocybin for depression (*n =* 9) and MDMA for PTSD (*n =* 10) had the strongest evidence base, while fewer meta-analyses were available for LSD in alcohol use disorder (*n =* 2) and depression (*n =* 2), ayahuasca in depression (*n =* 2), and MDMA in autism spectrum disorder (*n =* 2). Psilocybin demonstrated large effect sizes in major depression (Hedges’ g ≈ 1.05), with some evidence of sustained benefits up to six months. MDMA showed very large effects in reducing PTSD symptoms (Hedges’ g ≈ 1.24), often after 2–3 sessions. LSD yielded short-term benefits for alcohol use disorder (OR ≈ 2.0), though effects declined over time. Across studies, adverse events were generally mild and transient, with no consistent signal for serious harm. Considerable methodological variability was observed, including small and sometimes overlapping samples, heterogeneity, risk of bias, and limited long-term data. These constraints should be taken into account when interpreting the overall findings. **Conclusions**: Current evidence supports the short-term efficacy and safety of psychedelic-assisted therapy for selected psychiatric disorders, particularly depression and PTSD. However, the low methodological quality of studies and most meta-analyses, as well gaps in long-term safety data highlight the need for high-quality studies.

## 1. Introduction

Psychedelics are a group of psychoactive substances that are gaining renewed interest in medicine, especially in psychiatry. Their common feature is the psychedelic effect consisting in changing the sense of time and space, experiencing transcendental, mystical experiences so-called “ego dissolution” or a sense of unity with the universe, changing or sharpening sensory perception. The exact mechanism of therapeutic action is not clear. It has been postulated that it may be related to new self-perception, “ego dissolution”, as well as an expanded emotional spectrum and a change in the way of thinking [1]. These experiences, in turn, are associated with a reduction in depressive and anxiety symptoms, and an improvement of interpersonal relationships and overall functioning. Many studies on psychedelic substances focus on major depressive disorder (MDD), which is a mood disorder characterized by a persistent low mood, anhedonia, cognitive dysfunction, and significant impairment in daily functioning. Treatment-resistant depression (TRD) refers to an inadequate response to at least two antidepressant trials at and adequate dose and duration. Given the substantial disease burden associated with MDD and TRD, an exploration of novel therapeutic strategies such as psychedelic-assisted therapy has become increasingly important. It seems, therefore, that psychedelics can be helpful for a wide variety of mental problems, such as post-traumatic stress disorder (PTSD), alcohol use disorder (AUD), or autism spectrum disorder (ASD). So far, studies have shown that 3,4-Methylenedioxymethamphetamine (MDMA)-assisted therapy is highly effective in the treatment of PTSD [2]. The same applies to the use of psilocybin [3] and ayahuasca [4] in the treatment of major depression, including treatment-resistant depression. Beneficial effects have also been observed in neurodevelopmental disorders [5] or addictions [6]. However, it is worth noting at this point that the history of their use for medicinal purposes dates back thousands of years. Psilocybin and ayahuasca were (and still are) used by the indigenous peoples of Central and South America in religious rites and spiritual and medicinal rituals. However, for the last few decades, these sub-stances were classified as narcotic and illegal. The ban on their use also applied to medical use. Clinical trials, which began in the 1970s, were discontinued and banned for several decades. In the last few years, however, there have been scientific reports indicating their potential usefulness in the treatment of mental disorders, especially in patients who do not respond to standard treatment. The encouraging results of modern RCTs have thus initiated a new chapter in the attempt to apply them to psychiatry. The sudden increase in interest in this topic in the last few years is indicated by the growing number of scientific publications on the subject. It is currently also a hot topic in scientific conferences. Interestingly, there are far more review articles and meta-analyses than the original studies themselves. Some might say that psychiatrists and psychologists have struggled with this novelty. Therefore, it raises a warning to approach the quality of scientific evidence more carefully and coolly, carefully verifying its reliability. Despite the relatively large number of RCTs and meta-analyses, and undoubtedly positive results, psychedelic-assisted therapy is still not a registered treatment method. It remains an experimental option (with the exception of psilocybin, which was approved to treat depression under special conditions in Australia in 2023). The evidence accumulated to date is characterized by significant methodological and clinical variability, including differences in sample composition, concomitant medication use, dosing strategies, diagnostic definitions, and follow-up duration. Additional concerns relate to functional unblinding, given the distinct subjective effects of psychedelic substances, which complicate rigorous masking procedures. Furthermore, the strength and consistency of the evidence differ markedly across disorders: while psilocybin in depression and MDMA in PTSD cases is supported by comparatively stronger randomized data, the findings for LSD, ayahuasca, and other substances remain preliminary. These factors highlight the need for a cautious interpretation of the existing results and underscore the importance of further high-quality trials to establish long-term safety, durability of effects, and the clinical contexts in which these therapies may be most appropriate.

As numerous RCTs and meta-analyses do not provide a clear answer regarding the benefits and harms of psychedelics in the treatment of mental disorders, we aimed at con-ducting an umbrella review of the meta-analyses of RCTs that report on the efficacy and safety of psychedelic-assisted therapy for mental disorders.

## 2. Materials and Methods

### 2.1. Study Design

This study design followed an umbrella review framework, synthesizing evidence from previously published meta-analyses of randomized controlled trials. This review was conducted according to a pre-prepared, unregistered protocol.

### 2.2. Literature Search Strategy

We followed the guidelines according to PRISMA (Figure S1). In February 2025, the following databases were searched for systematic reviews with meta-analyses of randomized controlled trials (RCTs): PubMed, Scopus, and Web of Science. The search was performed by two independent groups of investigators. The eligible papers, their quality, and selected data were assessed.

The search query was as follows: (“psychedelic” OR “mescaline” OR “lysergic acid diethylamide” OR “LSD” OR “psilocybin” OR “ayahuasca” OR “N,N-dimethyltryptamine” OR “dimethyl-tryptamine” OR “DMT” OR “methylenedioxymethamphetamine” OR “MDMA” OR “3-Meo-DMT”) AND (“systematic review” OR “meta-analysis”). Searches were not limited by any filters, e.g., study type, language, or publication data. A manual search of the reference lists of included reviews was performed in addition to the digital search to ensure that no relevant articles were missed. Disagreements were resolved by discussion and consensus.

### 2.3. Study Selection

This umbrella review used the PICOS framework to develop detailed inclusion/exclusion criteria, as shown in Table 1. Based on this model and additional information, the following criteria of inclusion were developed: (1) agreeing with PICOS and (2) publications in English. The excluded criteria were as follows: (1) not confirmed with PICOS; (2) not an original article; (3) not in English; (4) full text was not available; and (5) unpublished.

Two reviewers independently screened articles by title, abstract, and then full text. Discrepancies were resolved through the additional consultation of a senior reviewer.

Systematic reviews that included both randomized and non-randomized evidence were eligible only if they contained at least one RCT relevant to the outcome of interest. In such cases, only RCT-derived data were extracted and analyzed, consistent with the standardized umbrella review methodology. Reviews containing exclusively non-randomized studies were excluded.

### 2.4. Data Extraction

Data related to the effects of psychedelics on mental disorders were extracted independently by two reviewers using a tailored form. The form included authors, year of publication, country, study design, number of studies included in the meta-analysis, type of psychedelics, diagnosis, and outcomes regarding efficacy, dosage used, and duration of treatment, as well as risks and safety. To ensure consistency in data extraction across reviews, we applied a predefined hierarchy for selecting outcomes from each meta-analysis. Priority was given to (1) the primary clinical efficacy endpoint (e.g., depression severity scales, CAPS for PTSD); (2) remission or response rates, when available; (3) standardized mean differences or effect sizes convertible to Hedges’ g; and (4) safety outcomes reported in a comparable format. When multiple eligible measures were provided within a single meta-analysis, we extracted the outcome with the most complete reporting and highest methodological relevance. Adverse events were classified using a unified framework consistent with the clinical trial methodology, distinguishing among (1) any adverse event, (2) treatment-emergent adverse events, and (3) serious adverse events. When inconsistent reporting prevented quantitative pooling, adverse events were summarized using a structured, itemized narrative to improve comparability across studies.

### 2.5. Quality Assessment

To ensure the quality of this umbrella review, the following assessment tools were used: the Assessment of Multiple Systematic Reviews 2 (AMSTAR2) and GRADE. AMSTAR2 provides a comprehensive assessment of systematic reviews and meta-analyses from analyses from several perspectives, including literature search, statistical analysis, bias, and conflict of interest. It is the most widely used tool for assessing the methodological quality of meta-analyses worldwide and has been used to assess meta-analyses including RCTs. The quality and certainty of evidence were evaluated by two teams of researchers using AMSTAR2 and GRADE (the evidence marked as high, moderate, low, or very low), respectively (Table 2).

### 2.6. Statistical Analysis

In the selected studies, effect sizes were converted to Hedges’ g to ensure consistency and comparability across studies. This standardized metric accounts for small sample size bias and allows for the integration of the results derived from various statistical approaches (e.g., standardized mean differences, t-values, or Cohen’s d). By transforming all outcomes into Hedges’ g, the meta-analytic synthesis maintains methodological rigor and uniformity in effect size interpretation. Quantitative synthesis was conducted only when sufficient and methodologically comparable data were available, allowing effect sizes or summary statistics to be harmonized across meta-analyses. In several areas, particularly for safety outcomes and secondary endpoints, data were incomplete, reported using non-comparable measures, or lacked the statistical information required for effect-size conversion. In these cases, a structured narrative synthesis was applied to avoid producing potentially misleading pooled estimates. To ensure an appropriate level of clinical utility for each mental disorder, the existing evidence was summarized into several best-documented recommendations.

To minimize the risk of double-counting participants from overlapping primary trials, we systematically screened all included meta-analyses for shared RCTs by comparing trial identifiers, study populations, and registration numbers. When the same RCT appeared in more than one meta-analysis, effect sizes were extracted from the most comprehensive or methodologically rigorous review or from the review providing the largest set of complete RCT data. When numerical disentanglement was not feasible, overlapping data were synthesized qualitatively to avoid inflation of precision.

Heterogeneity across meta-analyses was assessed using the I^2^ statistic and the χ^2^ test. When I^2^ exceeded 50% or when clinical or methodological differences suggested meaningful diversity between studies, random-effects models were applied. Fixed-effect models were used only in datasets with low heterogeneity. In cases where pooling was deemed inappropriate due to substantial heterogeneity (e.g., differences in dosing regimens, diagnostic criteria, or population characteristics), the results were synthesized narratively following a structured approach. Analyses were performed using RevMan 5.4 software and STATA 18.

## 3. Results

### 3.1. Search Results

**Figure 1:**
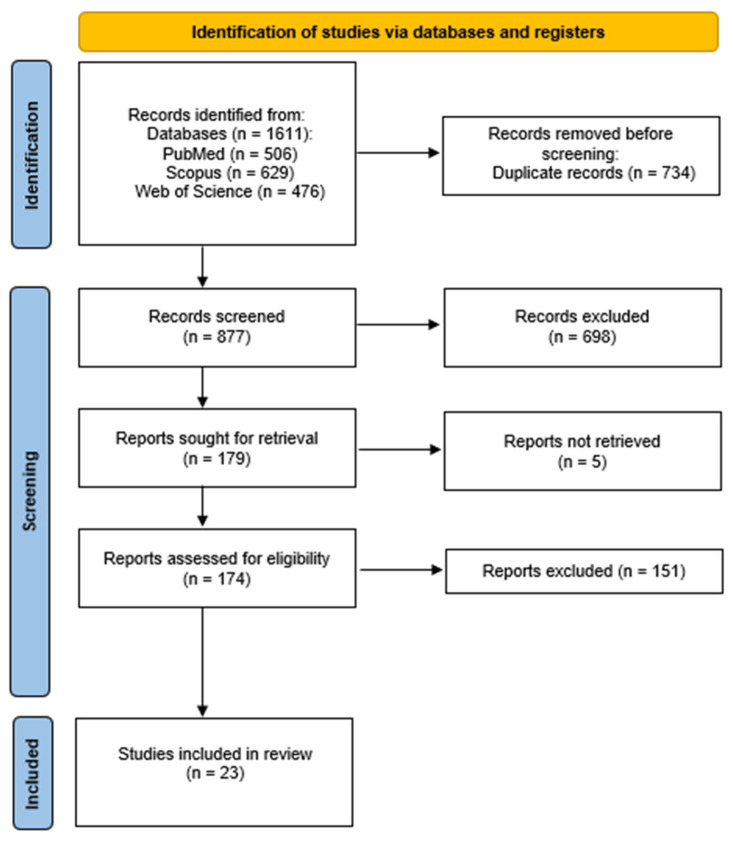
Flowchart showing an overview of the study selection process.

Following a comprehensive search of three databases, a total of 1611 articles were obtained. Following the removal of duplicates, the number of articles was reduced to 877. Following a thorough review of the titles and abstracts, 179 articles were selected for full-text evaluation. The full texts of the 176 potentially relevant articles were then downloaded and evaluated in full. Following this process, a total of 23 meta-analyses that satisfied the specified criteria were selected for inclusion in the systematic review. This process is described in the PRISMA flowchart (Figure 1). Selected meta-analyses have been grouped in Table 2 depending on the psychiatric disorder studied.

As an umbrella review aims to synthesize and evaluate evidence to inform the potential clinical utility of interventions, three key therapeutic areas were identified and examined based on the gathered data: the use of psilocybin in major depression (MD) cases, MDMA in post-traumatic stress disorder (PTSD) cases, and LSD in alcohol use disorder (AUD) cases. Each of these areas was selected due to the growing body of evidence supporting their efficacy and safety profiles, as well as their relevance to the current clinical research and potential future treatment frameworks. The review explores the strength of the evidence, treatment outcomes, and safety considerations for each compound within its respective clinical context.

The umbrella review included 23 meta-analyses that evaluated the clinical utility of psychedelic-assisted therapies across three key indications. A summary of the results of this work is presented in Tables S1–S4 (Supplementary Materials). Overall, the methodological quality of the included reviews was generally low, as reflected by the AMSTAR2 ratings, while the certainty of evidence (GRADE) varied from low to moderate.

In the area of psilocybin for depression, nine meta-analyses assessed its efficacy, safety, dose–response relationships, and administration (Table S1). All were rated as low quality according to AMSTAR2, except for the studies by Salvetti et al. (2024) [10] and Perez et al. (2023) [8], which provided moderately graded evidence regarding dosing and clinical efficacy. The GRADE certainty of evidence ranged from low to moderate, with moderate ratings typically assigned where pooled analyses and consistent outcomes supported the antidepressant effect of psilocybin, especially in the short-term follow-up.

For MDMA-assisted therapy in PTSD cases, ten meta-analyses were included (Table S2). While most were rated as low or critically low on the AMSTAR2 scale, a few demonstrated higher methodological rigor (Table 2). These reviews also yielded moderate certainty of evidence (GRADE), particularly in relation to symptom reduction, response and remission rates, and short- to mid-term safety profiles. However, many reviews either lacked pooled safety analyses or had limitations in assessing long-term adverse effects. Notably, Colcott et al. (2024) provided the most comprehensive and methodologically sound assessment of adverse events across both Phase 2 and Phase 3 trials [24].

In the case of LSD for alcohol use disorder, two reviews [6,26] supported the short-term efficacy of LSD in increasing abstinence and reducing alcohol consumption (Table S3). Both were rated as low quality (AMSTAR2) and had low certainty of evidence (GRADE), largely due to methodological concerns. Despite this, both reviews indicated a consistent short-term therapeutic effect. 

### 3.2. The Use of Psilocybin in the Treatment of Major Depression—Efficacy

**Figure 2:**
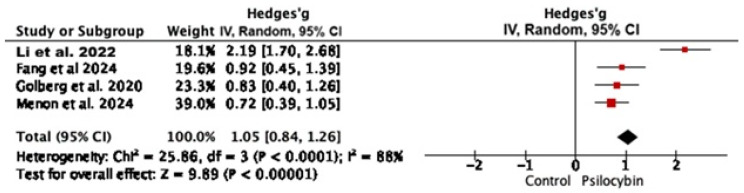
Hedges’ g for meta-analyses regarding psilocybin-assisted therapy for major depression [3,9,13,14].

Four meta-analyses provided sufficient data to be meta-analyzed. Data are depicted in Figure 2. Pooled findings confirm that psilocybin and psychedelic-assisted therapy significantly reduce depressive symptoms—Hedges’ g =1.05, CI: [0.84; 1.26], *p* < 0.001.

### 3.3. The Use of Psilocybin in the Treatment of Major Depression—Dose and Session Frequency

Most psilocybin studies for depression utilized moderate to high doses, typically in the range of 10–30 mg. When normalized to a 70 kg adult, these doses range from approximately 0.14 to 0.5 mg/kg. This reflects common clinical protocols. Only one study used strictly body-weight-adjusted dosing. Moderate doses are most common, balancing efficacy and tolerability. Table 3 summarizes the psilocybin doses in major depression.

According to Swieczkowski et al. (2025), doses of 0.215 mg/kg and 25 mg were particularly effective, with 25 mg outperforming 10 mg (RR—risk ratio = 5.62) [7]. Similarly, Perez et al. (2023) reported a significant dose–response relationship (*p* < 0.0001), with an estimated ED50 of 8.23 mg/70 kg and ED95 of 24.68 mg/70 kg [8]. Li et al. (2022) [3] found that high doses (30–35 mg/70 kg) showed greater efficacy (Hedges’ g = 3.059). The short-term subgroup (≤1 month) displayed stronger effects (Hedges’ g = 1.534) compared to long-term treatment (Hedges’ g = 1.123) [3]. Ko et al. (2023) [11] analyzed various psychedelics and found a significant reduction in depressive symptoms from Day 1 (SMD—standardized mean difference = −1.36) to Week 5 (SMD = −3.12), with the strongest effect observed in Weeks 3–5. However, the effect was no longer significant by Weeks 6–8 (SMD = −1.52; *p* = 0.14), suggesting a diminishing impact over time [11]. Menon et al. (2024) demonstrated superiority over control conditions at all evaluated timepoints: Day 2 (SMD = −1.11), Day 7 (SMD = −0.72), Day 14 (SMD = −0.67), and Day 42 (SMD = −0.56) [13].

Follow-up durations in the studies ranged from 2 weeks to 12 months, mostly covering short to medium terms. These findings highlight the nuanced efficacy of psilocybin in varying contexts and dosages, particularly its pronounced short-term benefits in alleviating depressive symptoms. However, the durability of these effects over extended periods remains uncertain. Furthermore, comprehensive research is needed to delineate its optimal therapeutic window and to address variability in individual responses. These considerations are critical as psilocybin therapy continues to evolve as a potential standard of care for major depressive disorder.

### 3.4. The Use of Psilocybin in the Treatment of Major Depression—Safety

Psilocybin was associated with a higher risk of adverse events, though no significant increase in serious adverse events was observed. Common short-term adverse events included headache, anxiety, nausea, and confusion [8,9,13]. These findings highlight psilocybin’s significant short-term antidepressant effects and manageable side effects, despite needing further study for long-term safety. Data variability prevented meta-analysis.

### 3.5. The Use of MDMA in the Treatment of PTSD—Efficacy

**Figure 3:**
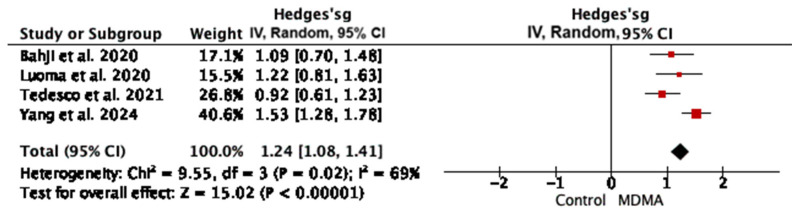
Hedges’ g for meta-analyses regarding MDMA-assisted therapy in PTSD cases [2,20,23,25].

MDMA-assisted psychotherapy has been evaluated in numerous studies and meta-analyses, consistently demonstrating strong therapeutic effects in the treatment of PTSD. Four meta-analyses provided sufficient data to conduct data synthesis. The results are depicted in Figure 3.

MDMA significantly reduced PTSD symptoms compared to the placebo or active placebo, with a large effect size (Hedges’ g = 1.24 95% CI: 1.08, 1.41, *p* < 0.001).

### 3.6. The Use of MDMA in the Treatment of PTSD—Dose and Session Frequency

PTSD studies using MDMA-assisted therapy report moderate to high doses, typically in the range of 75–125 mg. While some studies use body-weight-based dosing, most apply fixed-dose regimens. For a 70 kg adult, this corresponds to approximately 1.0–1.8 mg/kg. The consistency of dosing across studies reflects established therapeutic protocols. There is a limited exploration of dose–response curves in meta-analyses. Table 4 summarizes the MDMA doses in PTSD cases.

Yang et al. (2024) focused on clinical efficacy and dosing and found that high-dose MDMA (125 mg) was more effective than lower doses (e.g., 75 mg) [2]. On the other hand, Illingworth et al. (2021) [19] reported significant reductions in PTSD symptoms at both 75 mg (MD = −46.90; *p* < 0.00001) and 125 mg (MD = −20.98; *p* = 0.002) when compared to the active placebo. Analyzed studies clearly reported using it for 2 to 3 sessions, in line with the therapeutic protocols. Regarding the follow-up data—one long-term follow-up study (17–74 months) reported lasting relief in most participants, with only two cases of relapse [19]. Tedesco et al. (2021) supported these findings, showing that improvement was sustained up to 32 months (SMD = 0.81) [23].

### 3.7. The Use of MDMA in the Treatment of PTSD—Safety

Colcott et al. (2024) reported that MDMA-assisted therapy was associated with an increased likelihood of experiencing any adverse event during both the medication session (OR—odds ratio = 1.67) and the following week (OR = 1.59), with an even greater risk in Phase 3 trials (OR = 3.51) [24]. Adverse events were more frequent with higher doses and included jaw clenching, muscle tightness, nausea, appetite loss, chills, jitteriness, and blurred vision. Most reported adverse events were mild and resolved by 6 months; one serious event (suicidal ideation) was noted as potentially drug related. Importantly, there were no significant differences between groups in the incidence of treatment-emergent adverse events (RR = 1.03) or serious adverse events (RR = 0.95) [18]. Data variability prevented meta-analysis.

### 3.8. The Use of Psychedelics in the Treatment of Substance Use Disorder

Sicignano et al. (2024) and Krebs et al. (2012) both provide evidence that LSD-assisted therapy can significantly improve outcomes related to alcohol misuse and abstinence, particularly in the short term [6,26]. Sicignano et al. (2024) found that, in randomized, double-blind, placebo-controlled trials, both LSD (OR = 1.99; 95% CI: 1.10–3.61) and psychedelics generally (OR = 2.16; 95% CI: 1.26–3.69) significantly increased the odds of achieving abstinence or reducing alcohol use [6]. Krebs et al. (2012) reported similar findings, with LSD rapidly improving alcohol misuse with improvement remaining significant up to 6 months in the follow-up (OR = 1.66) [26]. Overall, LSD-assisted therapy appears to offer meaningful short- and mid-term benefits for alcohol misuse, though long-term efficacy and safety require further study. Data were insufficient to perform a meta-analysis.

### 3.9. The Use of Psychedelics in the Treatment of Neurodevelopmental Disorder

We identified another area of mental disorders that may benefit from psychedelic therapy: neurodevelopmental conditions such as autism. MDMA has shown potential therapeutic effects, including improved social functioning, enhanced social bonds, and feelings of love and friendship [5,27]. These results are presented in Table S4 (Supplementary Materials). However, the data are currently insufficient to draw definitive conclusions or to conduct a meta-analysis.

## 4. Discussion

Psychedelics are psychoactive substances that alter perception, consciousness, emotions, and create a sense of unity with the universe. Therapeutically, psychedelics can provide deep insights, new self-perception, transcendental experiences, and an expanded emotional spectrum. The research shows that mystical experiences and emotional breakthroughs from psychedelics can improve overall functioning and reduce depression and anxiety symptoms.

This umbrella review of 23 meta-analyses assessed the efficacy and safety of psychedelic-assisted therapy for mental disorders. The analysis was warranted due to the limited number of primary studies (RCTs) and the abundance of meta-analyses, making final conclusions challenging. Additionally, for some substances (e.g., MDMA and LSD), several meta-analyses rely on the same or partially overlapping primary RCTs, resulting in a smaller effective number of unique studies than suggested by the number of reviews. In addition, the available evidence was based on a limited number of trials, most with small sample sizes and early-phase, exploratory designs, which constrains statistical power and the stability of pooled estimates.

The reasons why psychedelic-assisted therapies are not yet formally registered in any country are undoubtedly complex. However, a more structured body of knowledge facilitates the identification of potential areas that warrant further exploration. The primary conclusions of this study pertain to two molecules: psilocybin and MDMA. Their efficacy in treating MD and PTSD is substantiated by substantial evidence. Nonetheless, our evaluation has highlighted several issues that require further clarification. The mechanism of action of psychedelics also requires further explanation. Psilocybin is rapidly dephosphorylated to psilocin, which acts primarily as a high-affinity partial agonist at the 5-HT2A receptor [28]. The activation of cortical 5-HT2A receptors is thought to modulate glutamatergic transmission and to disinhibit pyramidal neurons, at least partly via the effects on GABAergic interneurons [29]. These neurobiological effects may contribute to enhanced neuroplasticity and may partially underlie the observed improvements in depressive symptoms [30]. Lysergic acid diethylamide (LSD) also acts as a potent agonist at 5-HT2A and 5-HT1A receptors, with additional partial activity at dopaminergic and adrenergic receptors, which is thought to contribute to broad alterations in sensory, cognitive, and affective processing [29]. Ayahuasca contains N,N-dimethyltryptamine (DMT), a 5-HT2A agonist, and β-carbolines such as harmine, which inhibit monoamine oxidase A and thereby potentiate serotonergic signaling [31]. 3,4-Methylenedioxymethamphetamine (MDMA), in contrast, primarily acts as a monoamine-releasing agent, leading to marked increases in extracellular serotonin, norepinephrine, and dopamine levels [32]. It also enhances oxytocin release, which has been proposed to facilitate emotional processing and strengthen the therapeutic alliance during psychotherapy sessions [33]. These combined neurochemical effects are believed to contribute to the therapeutic effects of MDMA observed in PTSD cases [34].

### 4.1. The Use of Psychedelics in the Treatment of Major Depression

Among the available research results on psychedelic-assisted therapy in major de-pression cases, psilocybin comes to the fore. This umbrella review with meta-analyzed data has shown that psilocybin is effective in MD cases (Hedges’ g = 1.05, 95% CI: 0.84; 1.26, I. = 88%, *p* < 0.001). Importantly, the therapeutic effect can last up to 6 months [35], after just one dose [16]. Psilocybin induces subjective experiences with mystical characteristics, such as an increased sense of oneness with the universe, transcendence of time and space, loss of self, and euphoria. Importantly and significantly, numerous observations confirm that mystical experiences are correlated with long-term improvements in well-being, psychosocial functions, and reductions in anxiety and depressive symptoms. There have been many inconsistencies around psilocybin dosage. Some researchers point to the therapeutic potential of even the lowest doses [36]. However, other meta-analyses indicated a clear relationship between therapeutic effect and dose. This controversy was compounded by the recreational use of psychedelic substances in various doses, as well as their use in the so-called “underground”. Available data were too heterogenous to be meta analyzed, however the current evidence indicates that the therapeutic effect is dose-dependent, i.e., the higher the dose, the better the therapeutic outcomes. Effective doses for major depression are in the range of 25–36/70 kg, with higher doses required for treatment-resistant depression [3,8]. The optimal therapeutic dose, beyond which no further therapeutic gain is observed, depends on the specific patient population and potential confounding factors, like age and previous psychedelic experience. In general, the more severe and drug-resistant depression, the higher the doses required. Yet, another issue remains the administration schedule. According to Perez et al. (2023) [8], no significant difference between single- and two-dose psilocybin administration was observed. Moreover, the therapeutic effects of psilocybin in treating depression can be seen within a day, unlike antidepressants [8]. Only ketamine works so quickly [37]. However, psilocybin, unlike ketamine, has at the same time low potential for addiction and physical dependence [38]. Furthermore, the efficacy of psilocybin as compared to esketamine seemed to be similar with a better safety profile [39].

The Canadian Network for Mood and Anxiety Treatments (CANMAT) Task Force in a 2023 report summarized the results on psychedelic-assisted therapy for major depression [40]. CANMAT suggests psilocybin may effectively treat major depression, however the strength of the evidence has been described by this organization as weak (evidence level C). Thus, psilocybin remains an experimental option, available only through clinical trials or special programs. Similar regulations have been introduced in Australia.

In addition to the challenge of registering a substance like psilocybin for medicinal use, there is also the issue of finding a legal manufacturer. Psilocybin, which is present in certain mushrooms, poses challenges for stable synthesis and large-scale production.

### 4.2. The Use of Psychedelics to Treat Post-Traumatic Stress Disorder (PTSD)

The recommended PTSD treatment includes cognitive behavioral therapy (CBT) and selective serotonin reuptake inhibitors (SSRIs) like sertraline or paroxetine. CBT with exposure therapy is effective for certain patients but demands significant time and therapist expertise. One in four patients quits this therapy mid-treatment. Pharmacotherapy has limitations, with only 40–60% of patients showing an improvement. There is significant interest in psychedelic-assisted psychotherapy, particularly with ketamine and MDMA for PTSD treatment. MDMA-assisted therapy, a hallucinogen and psychostimulant, has proven more effective at reducing PTSD symptoms compared to placebo, as measured by the clinician-administered PTSD Scale (CAPS). In this umbrella review, we found that MDMA significantly reduced PTSD symptoms compared to the placebo or active placebo, with a large effect size (Hedges’ g = 1.24 95% CI: 1.08, 1.41, *p* < 0.001). The difference in the effectiveness of the therapy—the size of the effect—was very large—nearly 2 times more effective than the standard of treatment used so far. The improvement lasted between 2 to 32 months. Moreover, this therapy has been deemed safe and well-tolerated by patients [23]. Apart from temporary side effects like bruxism, jaw clenching, anxiety, headache, nausea, and a brief loss of appetite, no serious or permanent effects have been reported. The effective dosage ranges from 40 mg to 187.5 mg. In general, the number of sessions and follow-up length are more consistently reported in MDMA studies compared to psilocybin studies. This may reflect the more advanced regulatory status of MDMA-assisted therapy in PTSD cases. This highlights a greater emphasis on long-term outcome monitoring in MDMA trials.

The U.S. FDA’s refusal of registration, as of 13 August 2024, raised eyebrows. The decision pertains to methodological issues in studies on psychedelic-assisted psychotherapy, suggesting these should be addressed differently than the current standards. One contentious issue was the lack of standardized procedures for MDMA therapy, with varying doses and regimens used across different centers. Functional unblinding was another problem, as most participants guessed correctly whether they received MDMA or a placebo. The FDA proposed using active placebos. They also expressed concerns regarding inadequate monitoring of side effects, insufficient laboratory check-ups, and unclear long-term addiction risks. Therefore, it is essential to establish a standardized protocol for MDMA-assisted psychotherapy that includes optimal dosing and safety measures.

The research suggests potential benefits of psychedelic therapy for treating alcohol abuse and neurodevelopmental disorders. Studies on using psychedelics to address alcohol misuse are in the early stages [6,26]. LSD-assisted therapy has shown statistically significant short-term improvements in alcohol misuse and abstinence outcomes, but efficacy appears to decline over time, with no significant benefits detected at the 12-month mark. Although the initial results are promising, the current evidence is insufficient to recommend this therapy. Similarly, the research on neurodevelopmental disorders [5,27] indicates further trials with standardized session reporting and addressing adverse events are needed to validate long-term effects.

### 4.3. Safety of Psychedelic-Assisted Therapy

Current evidence suggests that psychedelic treatment is generally well-tolerated [12,24]. Commonly reported adverse events in psychedelic-assisted therapy include nausea, headaches, and anxiety for various indications and substances used. No studies have reported any serious side effects. There was a temporary increase in heart rate and systolic and diastolic blood pressure. Longer-lasting side effects included headaches (following psilocybin and MDMA), fatigue, depressed mood, and anxiety (following MDMA). The most frequent side effect of psychedelics was headache. However, it remains essential to monitor the safety of therapy according to the established protocol, which includes performing blood pressure measurements, ECGs, and basic laboratory tests, often omitted in previous studies. Reporting quality of adverse events varies between studies and meta-analyses. Most adverse events data are not pooled quantitatively, and many reviews report narrative summaries only.

However, in this umbrella review, we observed that reporting of safety outcomes was fragmented across trials and meta-analyses. Definitions of adverse events varied, with some studies relying on spontaneous reports and others using structured assessments. Serious adverse events were infrequently reported and often insufficiently detailed to allow quantitative synthesis.

Beyond the randomized controlled trials and meta-analyses included in this umbrella review, a broader body of clinical and observational literature suggests that psychedelic-assisted therapies are not universally beneficial and that a subset of patients may experience deterioration or clinically significant adverse psychological effects [41,42]. Reported phenomena include exacerbation of anxiety or depressive symptoms, emergence or worsening of suicidal ideation, and, more rarely, psychotic or dissociative symptoms [41,42]. Although such events appear to be infrequent, they highlight the importance of conservative patient selection, careful screening, preparation and integration, and long-term monitoring of outcomes.

The regulatory process for a substance’s medicinal use, when it is on the illegal list, is complex. Predicting all scenarios and consequences of the final decision poses significant medical, legal, and social challenges. We may need to rethink the current paradigm as it no longer aligns with today’s regulatory system and health needs. Nevertheless, the primary focus should always be on ensuring patients have access to effective modern therapies.

### 4.4. Limitations

This umbrella review has several important limitations. At the level of the primary literature, the evidence is characterized by substantial heterogeneity in study design, dosing protocols, treatment settings, outcome definitions, and adverse event reporting, as well as an uneven maturity of evidence across diagnostic categories. Most trials are small, include highly selected populations, and provide limited long-term follow-up, which restricts the generalizability and durability of observed effects. Limitations specific to this umbrella review include the absence of formal protocol preregistration, limited pooling restricted to methodologically comparable data, and the risk of overlapping primary trials across meta-analyses. Although steps were taken to mitigate these issues, they reduce the independence and precision of pooled estimates. Consequently, conclusions regarding efficacy and safety should be interpreted with caution.

## 5. Conclusions

The use of psychedelics in the therapeutic contexts has emerged as a promising avenue to address complex mental health conditions. The findings from this umbrella review collectively underscore the dynamic impact of psychedelic treatments on psychiatric conditions. The variability in observed effects across different studies reflects the complexity of treatment outcomes, influenced by factors such as dose, duration, and population characteristics. The research suggests that psychedelic-assisted therapy could treat PTSD, depression, addiction, or neurodevelopmental disorders. Notably, psilocybin-supported therapy for depression and MDMA-assisted psychotherapy for PTSD have shown substantial, lasting benefits with a good safety profile. However, past studies had methodological issues, requiring cautious interpretation. Despite this, the therapeutic potential of psychedelics is significant. Moving forward, it is crucial to introduce these substances into medicine with strict safety standards, uniform protocols, and long-term monitoring. Rather than more studies, quality randomized, blinded trials are needed to address side effects and abuse risks post-therapy. Addressing these unresolved topics may unlock greater global acceptance of psychedelic-assisted therapies.

## Figures and Tables

**Table 1:** PICOS framework.

**Patients**	Patients aged 18–65 years old with a diagnosis of any mental disorder (including MDD, MD, BD, PTSD, SUD, and other mental disorders) defined by the Research Diagnostic Criteria, Diagnostic and Statistical Manual of Mental Disorders (DSM-IV or DSM-5), or International Classification of Diseases (ICD-10, ICD-11)
**Intervention**	Psychedelics substances (mescaline, lysergic acid diethylamide, psilocybin, ayahuasca, dimethyltryptamine, and methylenedioxymethamphetamine)
**Comparator**	Placebo, active placebo
**Outcome**	Efficacy—clinical scales, response, and remission rates Safety, tolerability, and acceptability
**Study**	Meta-analyses of randomized controlled trials (RCTs) with a parallel group or crossover designs, relating to the efficacy or safety of any of the psychedelics

MDD—major depressive disorder; MD—major depression; BD—bipolar disorder; PTSD—post-traumatic stress disorder; SUD—substance use disorder; DSM-IV/DSM-5—Diagnostic and Statistical Manual of Mental Disorders; ICD-10/ICD-11—International Classification of Diseases; RCTs—randomized controlled trials.

**Table 2:** Selected meta-analyses grouped according to mental disorder.

Author, Year	k-Number of Studies	Type of Psychedelics	Diagnosis	Outcome	AMSTAR2	GRADE
Swieczkowski et al., 2025 [7]	3	psilocybin	MD	Efficacy, safety, dose	Low	Moderate
Li et al., 2022 [3]	5	psilocybin	MD	Efficacy	Low	Low
Perez et al., 2023 [8]	7	psilocybin	MD	Dose	Low	Moderate
Q. Fang et al., 2024 [9]	4	psilocybin	MD	Efficacy	Low	Low
Salvetti et al., 2024 [10]	12	psilocybin	MD	Route	Low	Moderate
Ko et al., 2023 [11]	7	LSD, ayahuasca, psilocybin	MD	Efficacy	Low	Moderate
Fang et al., 2024 [12]	8	psilocybin	MD	Efficacy, (safety without pool analysis)	Low	Low
Menon et al., 2024 [13]	6	psilocybin	MD	Efficacy, safety	Low	Low
Goldberg et al., 2020 [14]	4	psilocybin	MD	Efficacy	Low	Low
Aghajanian et al., 2024 [15]	7	psilocybin	MD	Efficacy	Low	Low
Leger et al., 2022 [16]	9	psilocybin, ayahuasca, LSD	MD	Efficacy	Low	Low
Hoskins et al., 2021 [17]	4	MDMA	PTSD	Efficacy	Critically low	Low
Shahrour et al., 2024 [18]	9	MDMA	PTSD	Efficacy, safety	Low	Moderate
Yang et al., 2024 [2]	7	MDMA	PTSD	Efficacy, safety, dose	Low	Moderate
Illingworth et al., 2021 [19]	4	MDMA	PTSD	Efficacy, safety, dose	Critically low	Low
Bahji et al.2020 [20]	5	MDMA	PTSD	Efficacy (safety, dose without pool analysis)	Low	Low
Amoroso et al., 2016 [21]	2	MDMA	PTSD	Efficacy (safety without pool analysis)	Low	Low
Bahji et al., 2023 [22]	7	MDMA	PTSD	Efficacy, (safety without pool analysis)	Moderate	Moderate
Tedesco et al., 2021 [23]	10	MDMA	PTSD	Efficacy (safety without pool analysis)	Low	Low
Colcott et al., 2024 [24]	8	MDMA	PTSD	Safety	High	Moderate
Luoma et al., 2020 [25]	5	MDMA	PTSD	Efficacy	Low	Low
Krebs et al., 2012 [26]	6	LSD	AUD	Efficacy, (safety without pool analysis)	Low	Low
Sicignano et al., 2024 [6]	4	LSD, psilocybin	AUD	Efficacy	Low	Low
Regan et al., 2021 [27]	27	MDMA	Sociability related outcomes, Autism	Efficacy	Low	Low
Kisely et al., 2023 [5]	14	MDMA	Social anxiety in Autism	Efficacy	Low	Low

MD—major depression; LSD—lysergic acid diethylamide; MDMA—3,4-Methylenedioxymethamphetamine.

**Table 3:** Doses of psylocibin in the treatment of major depression.

Original Dose	Dose Type	Mean Dose	Dose Category	Est. mg/kg (Assumed 70 kg)	Author
0.215/kg, 10 mg, 20 mg	mg/kg	10.07	Moderate	10.072	Świeczkowski et al., 2025 [7]
10–35 mg/70 kg	mg/kg	38.33	High	38.333	Li et al., 2022 [3]
10–30 mg	mg	20.00	Moderate	0.286	Perez et al., 2023 [8]
1–10 + 25 mg *	mg	12.00	Moderate	0.171	Q Fang et al., 2024 [9]

* Studies with doses of 1 mg, 10 mg, and 10 mg followed by 25 mg are included.

**Table 4:** Reported MDMA doses and estimated standardized values for selected meta-analyses.

Original Dose (mg)	Dose Type	Mean Dose	Dose Category	Est. mg/kg (Assumed 70 kg)	Author
75–125 MDMA prior to two or three sessions (12 sessions overall)	mg	70.67	Medium	1.010	Hoskins et al., 2021 [17]
50–125 (2–3 sessions)	mg	45.00	Low	0.643	Shahrour et al., 2024 [18]
25–125	mg	75.00	Medium	1.071	Yang et al., 2024 [2]
75–125	mg	100.00	High	1.429	Illingworth et al., 2021 [19]
50–125	mg	87.50	High	1.250	Bahji et al., 2020 [20]
50–180	mg	115.00	High	1.643	Bahji et al., 2023 [22]
50–187.5	mg	118.75	High	1.696	Tedesco et al., 2021 [23]
50–125	mg	87.50	High	1.250	Colcott et al., 2024 [24]

MDMA—3,4-Methylenedioxymethamphetamine.

## Data Availability

The data presented in this study are available upon request from the corresponding authors. The data are not publicly available due to privacy or ethical concerns.

## Supplementary Materials

The following supporting information can be downloaded at: https://www.mdpi.com/article/10.3390/jcm15010253/s1.

## Abbreviations

The following abbreviations are used in this manuscript:

RCT	randomized controlled trial
MDMA	3,4-methylenedioxymethamphetamine
LSD	lysergic acid diethylamide
PTSD	post-traumatic stress disorder
MD	major depression
AUD	alcohol use disorder
MDD	major depressive disorder
SUD	substance use disorder
BD	bipolar disorder
DSM	Diagnostic and Statistical Manual of Mental Disorders
ICD	International Classification of Diseases
PRISMA	Preferred Reporting Items for Systematic Reviews and Meta-Analyses
PICOS	Population, Intervention, Comparator, Outcomes, Study design
AMSTAR2	Assessment of Multiple Systematic Reviews 2
GRADE	Grading of Recommendations Assessment, Development and Evaluation
OR	odds ratio
RR	risk ratio
CI	confidence interval
SMD	standardized mean difference
ED50	effective dose for 50% of population
ED95	effective dose for 95% of population
MD	mean difference
SD	standard deviation
ECG	electrocardiogram
FDA	Food and Drug Administration
CBT	cognitive behavioral therapy
SSRIs	selective serotonin reuptake inhibitors
CANMAT	Canadian Network for Mood and Anxiety Treatments
CAPS	Clinician-Administered PTSD Scale
BDI	Beck Depression Inventory
SDS	Sheehan Disability Scale
PSQI	Pittsburgh Sleep Quality Index
DES	Dissociative Experiences Scale
ADR	adverse drug reaction
AE	adverse event
