# Supplementary material for: Efficacy and Safety of Psychedelics in Mental Disorder Cases: An Umbrella Review of Meta-Analyses of Randomized Controlled Trials
Source: J Clin Med. 2025 Dec 29;15(1):253. doi: 10.3390/jcm15010253 (PMC12786876; doi:10.3390/jcm15010253)
Supplement: Supplementary file 1 [file jcm-15-00253-s001.zip › jcm-4015010-supplementary.pdf]

Journal of Clinical Medicine

MDPI

Supplementary Materials for "Efficacy and Safety of Psychedelics in Mental Disorder Cases: An Umbrella Review of Meta-Analyses of Randomized Controlled Trials"

Table S1. Results of meta-analyses included in this umbrella review - major depression.

Author	Outcomes: 1) Efficacy related outcomes; 2) Dose and duration of treatment related outcomes; 3) Risk related outcomes
Święczkowski et al. 2025 [7]	1) By Day 8, pooled analysis showed a statistically better response to treatment with psilocybin compared to placebo (12.57 ± 3.88. vs. 06.25 ± 2.44; MD = 7.42; 95%CI: 10.07 to 4.78; p < 0.001). The improved response correlations were observed for measurements on Day 14 after treatment (16.11 ± 3.78 ± 6.02 ± 3.04; MD = 9.55; 95%CI: 12.44 to 6.65; p < 0.001). 2) Psilocybin 0.215mg/kg (RRs: 6.51; 95%CI: 1.61 – 11.29) and psilocybin 25 mg (RRs: 7.88; 95%CU: 5.15 – 10.57) were found to be more effective than placebo in the treatment of MDD basing on MADRS score at 8-days follow-up. Statistically significant differences were also observed between psilocybin 25 mg and psilocybin 10 mg (RRs = 5.62; 95%CI: 2.48 - 8.77). Psilocybin 10 mg (2.25; 95%CI: 0.33–4.75) did not show significant effects compared to placebo or psilocybin 0.215mg/kg ((2.25; 95%CI: 0.33–4.75) and (RRs: 4.21; 95%CI: 9.75 to 1.3) retrospectively). By Day 8, response to psilocybin was significantly better than placebo (12.57±3.88 vs. 6.25±2.44; MD = 7.42; 95% CI: 10.07 to 4.78; p < 0.001), with similar results on Day 14 (16.11±3.78 vs. 6.02±3.04; MD = 9.55; 95% CI: 12.44 to 6.65; p < 0.001). 3) Psilocybin was associated with a significantly higher risk of any adverse event compared to control (RRs = 1.43, CI: 1.119 to 1.73, p<0.001). Specifically, the risk of nausea was significantly higher with psilocybin (RRs = 8.35, CI: 3.26 to 21.41, p <0.001). No statistically significant differences in the context of serious AE (RRs=1.72, CI: 0.20 to 14.38, p=0.62), dizziness (RRs=5.83, CI: 0.74 to 46.09, p=0.09), headache (RRs=2.03, CI: 0.92 to 4.46, p=0.08) were observed.
Li et al. 2022 [3]	1) Primary depression: I2 =66.790%, Hedges' g=2.190, 95%CI= [1.423, 2.957], p<0.001. 2) Pool results for primary and secondary depression: High dose (30-35mg/70kg) manifested greater clinical efficacy (I2 <0.001%, Hedges' g=3.059, 95%CI= [2.269, 3.849], p<0.001). Interestingly, the antidepressive effect of psilocybin seemed to decline and then increase, when the dose increases in a certain range. Subgroups for duration included short-term (1 month) (I2 = 72.992%, Hedges' g = 1.534, 95% CI = [1.014, 2.054], p <0.001) and long-term (>1month) (I2 <0.001%, Hedges' g=1.123, 95%CI= [0.861, 1.385], p<0.001). Long-term subgroup showed a certain degree of remission. It is worth noting that the short-term subgroup showed more significant effect than long-term. 3) Pool results for primary and secondary depression: No significantly high incidence of adverse events was reported in all included studies.
Perez et al. 2023 [8]	2) For primary depression, a significant dose-response association was found (p< 0.0001), in presence of a considerable heterogeneity (I2= 80%) ED50= 8.23 mg/70kg;

 https://doi.org/10.3390/xxxxx

	ED95=24.68 mg/70kg (95IC: 19.29-48.73). There is a considerable level of heterogeneity with VPCs up to 95%. Effective doses per day were 24.68/70 kg for patients with primary depression. 3) Dose-response associations for various side effects, including physical discomfort, blood pressure increase, nausea/vomiting, headache/migraine, and the risk of prolonged psychosis.
Q Fang et al. 2024 [9]	1) A random effects model with six arms from four studies showed that psilocybin significantly reduced depressive scores compared to baseline conditions (SMD = 4.14, 95% CI = [1.86, 6.41], I 2 = 97 %, p < 0.01). Subgroup analysis with 10 plus 25 mg psilocybin also showed a significant reduction (SMD = 1.95, 95% CI = [1.41, 2.49], I 2 = 14 %, p = 0.31). A sensitivity analysis excluding the overlapping of subjects from Carhart-Harris et al., 2016 also showed statistical significance (SMD =4.42, 95% CI = [1.73, 7.12], I2 = 98 %, p < 0.01). 3) Three studies with 265 patients recruited in total reported adverse events. Headache (20.75 %), anxiety (13.58 %), nausea (12.08 %), and confusion (4.91 %), all transient and short-term, were reported in at least two studies. Only one study investigated drug adverse events longitudinally. Suicidal ideation (1.51 %), intentional self-injury (1.13 %), and hospitalization (0.38 %) were reported as serious adverse events from Day 2 to Week 3 since the treatment. Suicidal behavior (1.13 %) was observed from Week 3 to Week 12 after psilocybin use.
Salvetti et al. 2024 [10]	1) Random-effect meta-analysis showed that psilocybin significantly reduces patients' depressive symptoms severity compared to baseline (z = 12.11, p < 0.001, d = 2.14, 95% CI [2.48; 1.78]. Random-effect meta-analysis showed that psilocybin significantly reduces subjects' depressive symptoms severity compared to control (z = 3.60, p < 0.001, d = 2.37, 95% CI [3.66; 1.08]. 3) Psilocybin significantly reduces patients' depressive symptoms severity compared to baseline (z = 12.11, p < 0.001, d = 2.14, 95% CI [2.48; 1.78]. Such an effect was observed not only when single-dose psilocybin was administered (k = 6), but also when two-dose psilocybin was delivered (k = 7). Although two-dose psilocybin treatment displayed a more pronounced effect size compared to single-dose intervention (d = 2.42, 95% CI [2.74; 2.11] versus d = 1.87, 95% CI [2.43; 1.30]), the test for subgroups difference was not statistically significant (X 2 (1) = 2.86, p = 0.09). Subgroup analyses were not conducted in the case of comparison with controls due to the low number of studies employing a control group/condition (k = 7).
Ko et al. 2023 [11]	1) Pool for all psychedelics: Standardized mean differences were calculated at day 1 (n = 4 studies; SMD = 1.36, 95% CI: 2.50 to 0.22; p = .02), week 1 (n = 3; SMD = 1.37, 95% CI: 2.41 to0.34; p = .009), weeks 3–5 (n = 3; SMD = 3.12, 95% CI: 6.19 to 0.04; p = .05), and weeks 6–8 (n = 3; SMD = 1.52, 95% CI: 3.55 to 0.51; p = .14), demonstrating a significant reduction of depressive symptoms at all timepoints with the exception of weeks 6–8.
Fang et al. 2024 [12]	1) MD Hedges' g= -0.92, 95% CI: -1.4 ~0.44, I^2 = 77.89%, p <0.01, MS Hedges' g= -0.88, 95% CI: -1.45-~0.32, I^2 = 48.52%, p =0.14). 3) The pooled RRs for PAT versus control interventions for the proportion of participants with any adverse event during the entire follow-up period was 1.20 (95% CI, 1.01–1.42; I2=43%; 4 RCTs. PAT was associated with a small but statistically significant increase in the risk of any adverse event. The

	pooled RRs for PAT versus control interventions was 1.78 (95% CI, 1.10–2.86; I2=52%; 4 RCTs; n=373) for headache, 4.10 (95% CI, 0.71–23.65; I2=82%; 4 RCTs, n=373) for nausea, and 6.52 (95% CI, 1.19–35.87; I2=0%; 3 RCTs; n=269) for dizziness. PAT was associated with a significantly higher risk for headache and dizziness; risk for nausea showed a non-significant trend towards elevation. CIs were wide for nausea and dizziness, indicating substantial imprecision.
Menon et al. 2024 [13]	1) In the main analysis, the reduction in depression scores at day 7 was significantly greater with PAT than with control interventions (SMD, 0.72; 95% CI, 0.95 to 0.49; 5 RCTs [24–28]; n=403). Heterogeneity was low (I2=17%). The pooled SMD for the difference in depression change scores at day 2 was 1.11 (95% CI, 1.70 to 0.52; I2=79%; 5 RCTSs [23–25, 27, 28]; n=323), at day 14 was 0.67 (95% CI, 0.91 to 0.43; I2=23%; 5 RCTs [24–28]; n=403), and at day 42 was 0.56 (95% CI, 0.91 to 0.21; I2=56%; 3RCTs [25, 26, 28]; n=321). PAT was superior to control interventions at all time points. Heterogeneity was moderate to high in the day 2 and 42 analyses. At day 7, the pooled RRs for PAT versus control interventions was 3.42 (95% CI, 2.35–4.97, I2=0%; 4 RCTs; n=373) for study-defined response rate and 3.66 (95% CI, 2.26–5.92, I2=0%; 4 RCTs; n=373) for study defined remission rate. PAT was significantly superior to control treatment in both regards; heterogeneity was low in both analyses.
Goldberg et al. 2020 [14]	1) Across three placebo-controlled studies effects were large Hedges (g = 0.82 to 0.83) and statistically significant.
Aghajanian et al. 2024 [15]	1) The results of the primary analysis revealed a large and clinically observable reduction (SMC: -1.24, 95%CI: -1.83 to -0.65) of depressive symptomatology in patients receiving psilocybin in addition to supportive therapy compared to baseline measurements. The decrease was also marked when compared to placebo (p-value = 0.032).
Leger et al. 2022 [16]	1) Meta-analysis showed significant, large positive effect sizes for measures of anxiety (Cohen's d=1.26) and depression (Cohen's d=1.38) overall. These positive effects were also significant at acute (1week) and extended (>1week) time points. No significant differences were observed between trials using different psychedelic agents (psilocybin, ayahuasca or LSD) however, a significant difference was observed in favor of trials with multiple dosing sessions. 3) No serious ADR were reported.

MD– mean difference; RRs– risk ratio; CI– 95% confidence interval; MDD – major depres-sive disorder; MADRS – Montgomery–Åsberg Depression Rating Scale; SMD – standard-ized mean difference; ED50 / ED95 – effective dose 50% / 95%; VPC – variance partition coefficient; AE – adverse event; PAT – psychedelic-assisted therapy; RCT – randomized controlled trial; SMC – standardized mean change; ADR – adverse drug reaction; LSD- ly-sergic acid diethylamide

Table S2. Results of meta-analyses included in this umbrella review - post-traumatic stress disorder.

Author	Outcomes: 1) Efficacy related outcomes; 2) Dose and duration of treatment related outcomes; 3) Risk related outcomes
Hoskins et al. 2021 [17]	1) Four studies were available for inclusion in a meta-analysis of reduction in PTSD symptoms for MDMA-assisted therapy versus placebo/MDMA active placebo-assisted therapy. The standard mean difference was 1.09(95% CI 1.60 to 0.58) and I2 = 0%. Test for overall effect Z=4.16, p<0.0001).
Shahrour et al. 2024 [18]	1) Meta-analysis showed that MDMA-AT led to a significant reduction in the CAPS-5 severity scores as compared to the control group (SMD 1.10, 95% CI: 1.62 to 0.59;3) The estimated heterogeneity among the studies was moderate (I2=61%). Our meta-analysis demonstrated that more patients in the MDMA-AT group exhibited significant responses as compared to patients in the control group (RR 1.59, 95% CI: 1.22, 2.08). Heterogeneity among the studies was found to be minimal (I2=17%). The remission rate was found to be significantly greater in the intervention group as compared to the control group (RR 2.32, 95% CI: 1.47 to 3.66) with minimal heterogeneity (I2=0%).
Yang et al. 2024 [2]	1) Compared with the placebo, MDMA-AP significantly attenuated PTSD symptoms, as measured by a reduction in CAPS (Hedges' g =-1.532, 95% CI -2.567 to -0.497, P = 0.004) and SDS scores (Hedges' g =-0.461, 95% CI -0.761 to -0.160, P = 0.003) from baseline. Although there were similar proportions of patients with a 10-point reduction in CAPS score in the MDMA-AP group compared with the placebo group (RR = 1.197, 95% CI 0.961 to 1.491, P = 0.108), the percentages of patients no longer meeting CAPS score criteria (RR = 1.661, 95% CI 1.237 to 2.232, P= 0.001) and achieving remission (loss of diagnosis and a total CAPS score 11) (RR = 2.587, 95% CI 1.434 to 4.667, P = 0.002) were significantly higher in the MDMA-AP group compared with the placebo group. 2,3). The reduction from baseline in CAPS score was significantly increased in the high-dose MDMA-AP group compared with the low-dose MDMA-AP group (Hedges' g= -0.956, 95% CI -1.523 to -0.390, P = 0.001). However, there was no significant improvement in depression symptom severity as measured on the self-reported BDI-II (Hedges' g = -0.724, 95% CI -2.308 to 0.861, P = 0.371), sleep quality as measured by the PSQI (Hedges' g = -0.875, 95% CI -1.938 to 0.187, P = 0.106), and symptoms of dissociation as measured by the DES-II (Hedges' g = -1.302, 95% CI -2.739 to 0.134, P = 0.076). Additionally, the proportion of patients with a >30 % decrease in CAPS-IV total score was significantly larger in the high-dose MDMA-AP group than in the low-dose MDMA-AP group (RR = 2.871, 95% CI 1.055 to 7.811, P = 0.039). However, there was no significant difference in the percentages of participants who no longer met the CAPS-IV PTSD diagnostic criteria between the high and low-dose MDMA-AP groups (RR = 1.766, 95% CI 0.744 to 4.190, P = 0.197). High-dose MDMA administration significantly increased the risk of jaw clenching or tight jaw compared to low-dose MDMA (RR = 2.901, 95% CI 1.129 to 7.454, P = 0.027). Other AEs, including anxiety, dizziness, fatigue, and headache, had similar incidence rates in the high and low-dose MDMA-AP groups. Moreover, the incidence rates of AEs were similar in the high and low-dose MDMA-AP groups during the 7 days of contact.

Illingworth et al. 2021[19]	1,2) When compared to active placebo, intervention groups taking 75 mg (MD -46.90; 95% (confidence intervals) CI -58.78, -35.02), 125 mg (MD -20.98; 95% CI -34.35, -7.61) but not 100 mg (MD -12.90; 95% CI -36.09, 10.29) of MDMA with psychotherapy, had significant decreases in CAPS-IV scores, as did the inactive placebo arm (MD -33.20; 95% CI -40.53, -25.87). A significant decrease in BDI when compared to active placebo (MD -10.80; 95% CI -20.39, -1.21) was only observed at 75 mg. 3) Compared to placebo, participants reported significantly more episodes of low mood, nausea and jaw-clenching during sessions and lack of appetite after 7 days.
Bahji et al. 2020 [20]	1) The overall RR for PTSD clinical response was significantly higher in the experimental group (RR = 3.47, 95% CI: 1.70, 7.06). The overall RR for PTSD remission was similarly higher in the experimental group (RR = 2.63, 95% CI: 1.37, 5.02). The overall SMD for the change in PTSD symptom scores pre-versus-post intervention was 1.30 (95% CI: 0.66, 1.94), indicating a large effect size. MDMA-assisted psychotherapy was found to last several months (follow-up 2-72 months) after the completion of the intervention: Pre-vs-Follow-Up: SMD = 1.10, 95% CI: 0.42, 1.78.
Amoroso et al.2016 [21]	1) The cumulative effect size for primary outcome measures calculated for MDMA-AP in this analysis was large (Hedges' g=1.17; SE=0.09; 95% CI 0.38–1.90; p=0.033). The cumulative effect size for secondary outcome measures calculated for MDMA-AP in this analysis was large (Hedges' g=0.87; 95% CI 0.01–1.79; p=0.049).
Bahji et al.2023 [22]	1) Effect MDMA on PTSD symptoms scores: SMD (k = 6): 0.95 [1.28, 0.62], response rate: (k = 6): 3.21 [1.78, 5.79], remission rate: (k = 6): 2.32 [1.53, 3.53]. Long-term outcomes: One study reported outcomes at 17 and 74 months for 16 participants. Results showed sustained symptomatic relief, with two participants experiencing a relapse. 3) For overall psychedelic-assisted therapy, no significant association found with retention in treatment (RR: 1.00 [0.96–1.04]), the overall number of dropouts (response rate: 0.88 [0.56–1.37]), or dropouts due to adverse events (response rate: 1.50 [0.38–5.89]).
Tedesco et al. 2021 [23]	1) The overall RRs for a clinical response was significantly higher in the experimental group (RRs = 3.10, 95% CI: 1.29, 7.45). Regarding remission, a random-effects meta-analysis showed an overall RRs for remission to also be higher in the experimental group (RRs = 2.96, 95% CI: 1.63, 5.39). MDMA-assisted psychotherapy also resulted in a significant reduction in PTSD symptoms, with an SMD of 0.93 for change in symptom scores from before to after treatment (SMD = 0.93, 95% CI: 0.51, 1.36). Furthermore, this reduction in symptoms was maintained in the extended follow-up periods which ranged from 2-32 months (SMD = 0.81, 95% CI: 0.40, 1.23).
Colcott et al. 2024 [24]	3) In Phase 2 studies, MDMA-AP was associated with increased odds of any side effect during medication sessions (OR=1.67, 95%CI (1.12, 2.49)) and in the 7 days following (OR=1.59, 95%CI (1.12, 2.24)) relative to control conditions. In Phase 3 studies, MDMA-AP was associated with increased odds of any adverse event during the treatment period relative to placebo-assisted psychotherapy (OR=3.51, 95%CI (2.76, 4.46)).
Luoma et al. 2020 [25]	1) MDMA effect on PTSD symptoms scores: effect size = Hedges g = 1.22. No significant difference in the overall effect size between MDMA and other classic psychedelics.

MDMA- 3,4-Methylenedioxymethamphetamine; CI- 95% confidence interval; MDMA-AT- MDMA-assisted psychotherapy; CAPS-5- Clinician-Administered PTSD Scale for DSM-5; DSM-5-

Diagnostic and Statistical Manual of Mental Disorders; SMD- standardized mean difference; RR- rate ratio; SDS- Sheehan Disability Scale; BDI-II- Beck Depression Invento-ry-Second Edition; PSQI- Pittsburgh Sleep Quality Index; DES-II- Dissociative Experiences Scale; CAPS-IV- Clinician Administered PTSD Scale for DSM-IV; AE- adverse events; MD- mean difference; BDI- Beck Depression Inventory; RRs- risk ratio; OR- odds ratio

Table S3. Results of meta-analyses included in this umbrella review - alcohol use disorder.

Author	Outcomes: 1) Efficacy related outcomes; 2) Dose and duration of treatment related outcomes; 3) Risk related outcomes
Sicignano et al 2024 [6]	1) At the first recorded follow-up, LSD [n = 3, OR 1.99 CI: 1.10 to 3.61)] and any psychedelic [n = 4, OR 2.16 (95%CI: 1.26 to 3.69)] enhanced the odds of patients achieving abstinence or a substantial reduction in drinking alcohol versus placebo. 3) One trial found an instance of suicidal ideation as well as transient increases in blood pressure.
Krebs et al.2012 [26]	1) The pooled odds ratio on improvement in alcohol misuse between the LSD and control groups was 1.96 (95% CI, 1.36–2.84; p = 0.0003) at the first reported follow-up. Among the five trials with dichotomized data, 185 of 315 (59%) LSD patients and 73 of 191 (38%) control patients were improved at the first reported follow-up, and the pooled benefit difference was 16% (95% CI, 8%25%; p = 0.0003), or, equivalently, the number needed to treat is six. At short-term follow-up (2–3 months post-treatment), three trials reported treatment response, and the pooled odds ratio between the LSD and control groups was 1.85 (95% CI, 1.14–3.00; p = 0.01). At medium-term follow-up (6 months post-treatment), five trials reported treatment response, and the pooled odds ratio between the LSD and control groups was 1.66 (95% CI, 1.11–2.47; p = 0.01). At long-term follow-up (12 months post-treatment), four trials reported treatment response, and the pooled odds ratio between the LSD and control groups was 1.19 (95% CI, 0.74–1.90; p = 0.47). Among the three trials that reported maintained abstinence from alcohol use, there was a beneficial effect of LSD at the first reported follow-up (1–3 months post-treatment) (OR, 2.07; 95% CI, 1.26–3.42; p = 0.004) and short-term follow-up (2–3 months post-treatment) (OR, 1.80; 95% CI, 1.07–3.04; p = 0.03), which was not statistically significant at medium-term follow-up (6 months posttreatment) (OR, 1.42; 95% CI, 0.65–3.10; p = 0.38)

LSD- lysergic acid diethylamide; OR- odds ratio; CI- 95% confidence interval

Table S4. Results of meta-analyses included in this umbrella review - neurodevelopmental disorder.

Author	Outcomes: 1) Efficacy related outcomes; 2) Dose and duration of treatment related outcomes; 3) Risk related outcomes
Kisely et al. 2023 [5]	1) There were small benefits of psilocybin for social anxiety in adults with autism.
Regan et al. 2021 [27]	1) The results of meta-analysis revealed a moderate-to-large effect (d = 0.86; 95% CI [0.68, 1.04]; r = .39; 95% CI [.32, .46]) of MDMA on self-reported sociability-related outcomes (e.g., feeling loving, talkative, and friendly).

CI- 95% confidence interval; MDMA- 3,4-Methylenedioxymethamphetamine

Section and Topic	Item #	Checklist item	Location where item is reported
**TITLE**			
Title	1	Identify the report as a systematic review.	Title
**ABSTRACT**			
Abstract	2	See the PRISMA 2020 for Abstracts checklist.	Abstract
**INTRODUCTION**			
Rationale	3	Describe the rationale for the review in the context of existing knowledge.	Introduction
Objectives	4	Provide an explicit statement of the objective(s) or question(s) the review addresses.	end of Introduction
**METHODS**			
Eligibility criteria	5	Specify the inclusion and exclusion criteria for the review and how studies were grouped for the syntheses.	Section 2.3
Information sources	6	Specify all databases, registers, websites, organisations, reference lists and other sources searched or consulted to identify studies. Specify the date when each source was last searched or consulted.	Section 2.2
Search strategy	7	Present the full search strategies for all databases, registers and websites, including any filters and limits used.	Section 2.2
Selection process	8	Specify the methods used to decide whether a study met the inclusion criteria of the review, including how many reviewers screened each record and each report retrieved, whether they worked independently, and if applicable, details of automation tools used in the process.	Section 2.3
Data collection process	9	Specify the methods used to collect data from reports, including how many reviewers collected data from each report, whether they worked independently, any processes for obtaining or confirming data from study investigators, and if applicable, details of automation tools used in the process.	Section 2.4
Data items	10a	List and define all outcomes for which data were sought. Specify whether all results that were compatible with each outcome domain in each study were sought (e.g. for all measures, time points, analyses), and if not, the methods used to decide which results to collect.	Table 1, Section 2.4
	10b	List and define all other variables for which data were sought (e.g. participant and intervention characteristics, funding sources). Describe any assumptions made about any missing or unclear information.	Section 2.4
Study risk of bias assessment	11	Specify the methods used to assess risk of bias in the included studies, including details of the tool(s) used, how many reviewers assessed each study and whether they worked independently, and if applicable, details of automation tools used in the process.	Section 2.5
Effect measures	12	Specify for each outcome the effect measure(s) (e.g. risk ratio, mean difference) used in the synthesis or presentation of results.	Table 1, Table 4, Section 2.6
Synthesis methods	13a	Describe the processes used to decide which studies were eligible for each synthesis (e.g. tabulating the study intervention characteristics and comparing against the planned groups for each synthesis (item #5)).	Section 3.1
	13b	Describe any methods required to prepare the data for presentation or synthesis, such as handling of missing summary statistics, or data conversions.	Section 2.6
	13c	Describe any methods used to tabulate or visually display results of individual studies and syntheses.	Section 2, Section 3
	13d	Describe any methods used to synthesize results and provide a rationale for the choice(s). If meta-analysis was performed, describe the model(s), method(s) to identify the presence and extent of statistical heterogeneity, and software package(s) used.	Section 2.6
	13e	Describe any methods used to explore possible causes of heterogeneity among study results (e.g. subgroup analysis, meta-regression).	Section 2.6
	13f	Describe any sensitivity analyses conducted to assess robustness of the synthesized results.	Section 2.6
Reporting bias assessment	14	Describe any methods used to assess risk of bias due to missing results in a synthesis (arising from reporting biases).	Section 2.6, Section 3
Certainty assessment	15	Describe any methods used to assess certainty (or confidence) in the body of evidence for an outcome.	Section 2.5, Section 2.6, Section 3
**RESULTS**			
Study selection	16a	Describe the results of the search and selection process, from the number of records identified in the search to the number of studies included in the review, ideally using a flow diagram.	Section 3.1, Figure 1
	16b	Cite studies that might appear to meet the inclusion criteria, but which were excluded, and explain why they were excluded.	Figure 1
Study characteristics	17	Cite each included study and present its characteristics.	Section 3
Risk of bias in studies	18	Present assessments of risk of bias for each included study.	Section 3, Table 2
Results of individual studies	19	For all outcomes, present, for each study: (a) summary statistics for each group (where appropriate) and (b) an effect estimate and its precision (e.g. confidence/credible interval), ideally using structured tables or plots.	Supplementary Materials
Results of syntheses	20a	For each synthesis, briefly summarise the characteristics and risk of bias among contributing studies.	Section 3
	20b	Present results of all statistical syntheses conducted. If meta-analysis was done, present for each the summary estimate and its precision (e.g. confidence/credible interval) and measures of statistical heterogeneity. If comparing groups, describe the direction of the effect.	Section 3
	20c	Present results of all investigations of possible causes of heterogeneity among study results.	Section 3, Section 4
	20d	Present results of all sensitivity analyses conducted to assess the robustness of the synthesized results.	Section 3
Reporting biases	21	Present assessments of risk of bias due to missing results (arising from reporting biases) for each synthesis assessed.	Section 3, Section 4
Certainty of evidence	22	Present assessments of certainty (or confidence) in the body of evidence for each outcome assessed.	Section 3
**DISCUSSION**			
Discussion	23a	Provide a general interpretation of the results in the context of other evidence.	Section 4
	23b	Discuss any limitations of the evidence included in the review.	Section 3, Section 4
	23c	Discuss any limitations of the review processes used.	Section 4
	23d	Discuss implications of the results for practice, policy, and future research.	Section 4
**OTHER INFORMATION**			
Registration and protocol	24a	Provide registration information for the review, including register name and registration number, or state that the review was not registered.	Section 2.1
	24b	Indicate where the review protocol can be accessed, or state that a protocol was not prepared.	Section 2.1
	24c	Describe and explain any amendments to information provided at registration or in the protocol.	Section 2.1
Support	25	Describe sources of financial or non-financial support for the review, and the role of the funders or sponsors in the review.	Funding
Competing interests	26	Declare any competing interests of review authors.	Conflicts of Interest
Availability of data, code and other materials	27	Report which of the following are publicly available and where they can be found: template data collection forms; data extracted from included studies; data used for all analyses; analytic code; any other materials used in the review.	Section 3, Supplementary Materials, Data Availability Statement

Figure S1. PRISMA checklist.

Disclaimer/Publisher's Note: The statements, opinions and data contained in all publications are solely those of the individual author(s) and contributor(s) and not of MDPI and/or the editor(s). MDPI and/or the editor(s) disclaim responsibility for any injury to people or property resulting from any ideas, methods, instructions or products referred to in the content.
